# The efficacy of mobile phone-based text message interventions (‘Happy Quit’) for smoking cessation in China

**DOI:** 10.1186/s12889-016-3528-5

**Published:** 2016-08-19

**Authors:** Yanhui Liao, Qiuxia Wu, Jinsong Tang, Fengyu Zhang, Xuyi Wang, Chang Qi, Haoyu He, Jiang Long, Brian C Kelly, Joanna Cohen

**Affiliations:** 1 Mental Health Institute of the Second Xiangya Hospital, Central South University. National Clinical Research Center on Mental Disorders & National Technology Institute on Mental Disorders. Hunan Key Laboratory of Psychiatry and Mental Health, Changsha, Hunan 410011 China; 2 Department of Sociology & Center for Research on Young People’s Health (CRYPH), Purdue University, 700 W State Street, West Lafayette, IN 47907 USA; 3 Institute for Global Tobacco Control (IGTC) at Johns Hopkins Bloomberg School of Public Health, 2213 McElderry St., Fourth Floor, Baltimore, MD 21205 USA; 4 Department of Psychiatry and Biobehavioral Sciences, UCLA, 760 Westwood Plaza, Los Angeles, CA 90095 USA

**Keywords:** Mobile phone-based text message, Interventions, ‘Happy Quit’, Smoking cessation, China

## Abstract

**Background:**

Considering the extreme shortage of smoking cessation services in China, and the acceptability, feasibility and efficacy of mobile phone-based text message interventions for quitting smoking in other countries, here we propose a study of “the efficacy of mobile phone-based text message interventions (‘Happy Quit’) for smoking cessation in China”. The primary objective of this proposed project is to assess whether a program of widely accessed mobile phone-based text message interventions (‘Happy Quit’) will be effective at helping people in China who smoke, to quit. Based on the efficacy of previous studies in smoking cessation, we hypothesize that ‘Happy Quit’ will be an effective, feasible and affordable smoking cessation program in China.

**Methods/Design:**

In this single-blind, randomized trial, undertaken in China, about 2000 smokers willing to make a quit attempt will be randomly allocated, using an independent telephone randomization system that includes a minimization algorithm balancing for sex (male, female), age (19–34 or >34 years), educational level (≤ or >12 years), and Fagerstrom score for nicotine addiction (≤5, >5), to ‘Happy Quit’, comprising motivational messages and behavioral-change support, or to a control group that receives text messages unrelated to quitting. Messages will be developed to be suitable for Chinese. A pilot study will be conducted before the intervention to modify the library of messages and interventions. The primary outcome will be self-reported continuous smoking abstinence. A secondary outcome will be point prevalence of abstinence. Abstinence will be assessed at six time points (4, 8, 12, 16, 20 and 24 weeks post-intervention). A third outcome will be reductions in number of cigarettes smoked per day.

**Discussion/Implications:**

The results will provide valuable insights into bridging the gap between need and services received for smoking cessation interventions and tobacco use prevention in China. It will also serve as mHealth model for extending the public health significance of other interventions, such as mental health interventions.

**Trial registration:**

NCT02693626 (Registration data April 11, 2016).

## Background

### Tobacco use and cigarette smoking in China

Tobacco use is the leading cause of avoidable morbidity and mortality in China (a country does not have a comprehensive tobacco control policy) [1, 2]. From 1880 to 2009, annual global consumption of cigarettes increased from an estimated 10 billion cigarettes to approximately 5.9 trillion cigarettes, with 38 % of the total consumption in China [3].

According to the Global Adult Tobacco Survey in 14 countries, more than half of men (52.9 % of men and 2.4 % of women) were current tobacco smokers in China [4, 5]. Tobacco smoking was responsible for about 673,000 premature deaths in Chinese adults who were 40 years of age or older in 2005 [1]. Total tobacco-attributable deaths will rise from 5.4 million in 2005 to 6.4 million in 2015 and 8.3 million in 2030 [6], while China’s annual tobacco-attributable deaths could rise to 2 million by the year 2025 [7]. On the basis of current consumption patterns, approximately 450 million adults will be killed by cigarette smoking between 2000 and 2050 [8]. These epidemiological studies demonstrate the serious nature of tobacco use in China and also highlight the need for smoking cessation interventions.

### Shortage of smoking cessation services in China

Smoking cessation rates are very low. Insufficient smoking cessation services are one of the most important contributing factors to the low cessation rates reported in China. Furthermore, clinical studies on smoking cessation remain inadequate in China [7]. A sample from 21 cities in China reported that almost half of smokers intended to quit, however, the prevalence of smoking cessation among those urban-based smokers was only 10.1 % [9].

Quitting rates were also low among Chinese physicians, and China’s medical community has not been sufficiently active in tobacco control [10]. The delivery of advice on quitting smoking was not common among health care providers. For example, a survey revealed that approximately 60 % of smokers were not asked about their smoking behaviours on their visit to a clinician, and approximately 67 % of them were not advised to quit smoking [11]. A survey showed that although the majority of nurses asked about smoking status, few assisted patients with quitting [12].

In order to minimize the huge gap between the need and demand for smoking cessation service, health-care professionals should shoulder leadership to provide smoking cessation services that are readily available, effective and cheap.

### Smoking cessation support delivered via mobile phone text messaging

The rapid development of mobile health technology (mHealth) provides unprecedented opportunities to improve health services and to reach underserved populations. Mobile phone based short text messaging service (SMS) has the potential to deliver smoking cessation support to large population groups, and the effectiveness of SMS programs to improve smoking cessation [13], which has been reported in other countries with different populations [14–18]. Because mHealth may be involved in how well and how quickly mobile phones are integrated into health care, and how well they serve the needs of the entire population, it deserves the attention of both the healthcare and public health community [19].

There are several potential benefits of SMS programs for smoking cessation: a) the easiness of use anywhere at any time; b) cost-effective delivery; c) scalability to large populations regardless of location; d) the ability to tailor messages to key user characteristics (such as age, sex, education); e) the ability to send time-sensitive messages with an ‘always on’ device; and f) the provision of content that can distract the user from cravings. The use of mobile phones for smoking cessation should be encouraged as they become even more ubiquitous and as technological advances increase the number of applications and functions available. Therefore, it is important to design mobile phone-based services that can be effective at helping people who smoke, to quit [14].

Importantly, mobile phone technologies are a way to reach a large proportion of the Chinese population. The number of mobile phone subscribers in China from February 2014 to February 2015 was about 1.29 billion (http://www.statista.com/). More than 90 % of Chinese use mobile phones; thus, it is a widely accessible technology that is highly suited to interventional purposes in China. Furthermore, the reading and sending of SMS messages are the most frequent activities when Chinese use a mobile phone. There is an urgent need to design such a population-based widely accessible smoking cessation program in China.

### Implications of our experiment

Tobacco use is a major cause of disease burden and the number one preventable cause of death in the world. About one-third of the world’s tobacco is produced and consumed in China. Among the general population of smokers, most report a desire to quit. Unfortunately, the availability of smoking cessation programs in China is extremely limited, and the majority of cessation attempts end in relapse. The rapid development of mobile health technology (mHealth) provides unprecedented opportunities to improve health services and to reach underserved populations, and the effectiveness of text message mobile phone programs to improve smoking cessation has been reported in other countries.

Considering the extreme shortage of smoking cessation programs in China, and the efficacy of mobile phone text message-based program for smoking cessation reported in other countries, we propose to design such a population-based widely accessible smoking cessation program in China. This proposed study is designed to determine the effectiveness of mobile phone-based text message interventions (‘Happy Quit’) for smoking cessation in China, which will be assessed by self-reported smoking abstinence at 4, 8, 12, 16, 20 and 24 weeks post-intervention. This proposed study has important implications in providing valuable insights into bridging the gap between need and services received for smoking cessation interventions and tobacco use prevention in China. It will also serve as a mHealth model for extending the public health significance of interventions, such as mental health interventions.

## Research design and methods

### Hypotheses and theoretical model

Primary Hypothesis: We hypothesize that ‘Happy Quit’ will be tested as an effective, feasible and affordable smoking cessation program in China. To be specific, the text messages intervention group will have higher rates of self-reported continuous smoking abstinence and point prevalence of abstinence when compared to control-group; and by assessing the number of cigarettes smoked per day, the text-based intervention will help those relapse smokers smoke fewer cigarettes than before. Secondary Hypothesis: Mobile phone-based text message interventions (‘Happy Quit’ program) for smoking cessation will have wider influence on smoking cessation in China.

**Fig. 1:**
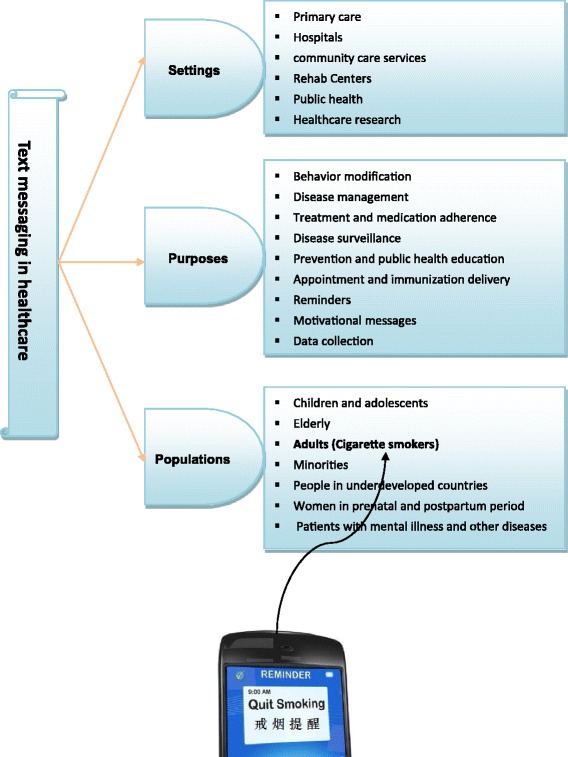
Text messaging in healthcare

**Fig. 2:**
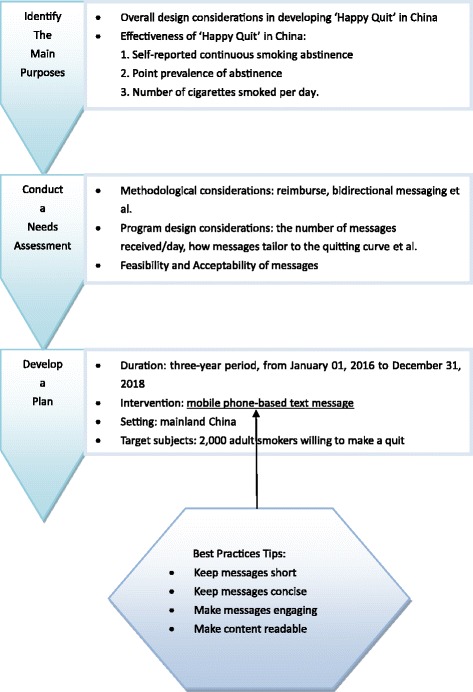
Worksheet for short message service (SMS) implementation planning

Theoretical model: Fig. 1 illustrates the theoretical model. In this model, text messaging could be applied in healthcare widely: settings range from primary care to community care services to public health; purposes from behavior modification to disease management, treatment and medication adherence to data collection; populations vary from children and adolescents to elderly, from cigarette smokers to Patients with mental illness and other diseases. Figure 2 Shows the worksheet for short message service (SMS) implementation planning for ‘Happy Quit’ program.

## Methods

### Setting and sample

This large, simple randomized trial of a new smoking cessation service will be conducted using mobile phone text messaging in China. A total of 2000 eligible participants will be selected randomly to the intervention or control groups.

Eligibility: daily smokers 18 years of age and older living in China.

Inclusion Criteria:

1. Daily Chinese cigarette smokers.
2. 18 years of age and older living.
3. Being able to read and write in Chinese.
4. Owning a text-capable cell phone and knowing how to text.
5. Willing to make an attempt to quit smoking in the next month.
6. Willing to provide informed consent to participate in the study.

Exclusion Criteria:

1. Nonsmokers
2. Below 18 years old.
3. Unable to read and write in Chinese.

No restrictions will be placed on location within mainland China. Given that this study will be mainly performed with text messaging, no restrictions will be placed on setting or location for confirmation of informed consent or for data collection.

### Procedures

We will advertise this service to smokers on the radio, bus billboards and online (e.g., websites, QQ, WeChat) and in newspapers, hospitals and pharmacies. Potential participants will register their interest by sending a text message. Research assistants then will telephone respondents to assess eligibility and collect baseline data.

In this single-blind, randomized trial, undertaken in China, smokers willing to make a quit attempt will be randomly allocated, using an independent telephone randomization system that includes a minimization algorithm balancing for sex (male, female), age (19–34 years, >34 years), educational level (to age ≤12 years, >12 years), and Fagerstrom score for nicotine addiction (≤5, >5), to a mobile phone text messaging smoking cessation program (‘Happy Quit’), comprising motivational messages and behavioral-change support, or to a control group that receives text messages unrelated to quitting. The system will automatically generate intervention or control group texts according to the allocation. Outcome assessors will be masked to treatment allocation.

Participants will be allocated to either a control group or to a group that receives the mobile-based smoking cessation program. All participants will be free to participate in any other smoking cessation service or support that they wish to use if possible and available. All Participants in both the intervention group and control group will be asked to set a quit date within one month of randomization.

Messages will be developed with the input of smokers and smoking cessation professionals. The intervention will include motivational messages and behavior-change techniques. Messages will encourage participants to persevere with the quit attempt and will focus on their success so far. The motivational messages will provide positive feedback and emphasize the benefits achieved by quitting and provide information about the consequences of smoking, how to quit and stay quit, and how others would approve of successful abstinence. The behavior change messages will prompt participants to get rid of cigarettes, ashtrays, and lighters and to avoid environments where they would normally smoke, and encourage participants to identify the challenges of quitting and plan how to overcome them. The messages also promote the use of nicotine replacement therapy if possible and available.

Participants who allocate to the intervention group will receive this program of interventions, which will be based around setting a quit date within 30 days of randomization. As described above, they will receive regular, personalized text messages providing smoking cessation advice, support and distraction. This content will also cover information relevant to quitting—for example, symptoms to expect on quitting, tips to cope with craving, tips to avoid weight gain and improve nutrition; advice on avoiding smoking triggers; instructions on breathing exercises to perform instead of smoking; motivational support (for example feedback on amount of money and life years saved) and distraction (for example, general interest, sports, fashion, trivia, travel). The messages will be developed by a multidisciplinary team including young adults, health related researchers and experts in cognitive behavioral therapy, and smoking cessation. An algorithm based on keyword matching will be developed to match participant characteristics (preferences, smoking history, barriers to cessation, etc.) with a database of over 1000 text messages so that an individualized program will be provided.

A quit day will be negotiated with each participant, and five messages will be sent per day for the time leading up to the quit day and the following 12 weeks. On the quit day, a free month of outgoing text messaging also begins, with participants encouraged to tell all their friends and family they are quitting on that day. This process will encourage communication and support, and also help with distraction, given the time that will spend in writing and receiving text messages. Several other text message based services will also be provided for the intervention group: Quit buddy (participants with similar characteristics and quit days will be put in touch with each other); text crave (participants could ‘pull’ text messages on demand by sending a text message to a short code number and they would receive a tip on how to get through their cravings); and TXT quizzes (questions will be sent out, following by answers the next day).

Twelve weeks after randomization, coinciding approximately with the end of the free text messaging month, the intervention will become much less intensive, with the number of sent text messages reduced from five per day to three per week until the end of the 24 week (that is, six month) follow up. These messages will focus on maintaining cessation among those who had quit. Control group participants only will receive one text message every week, thanking them for being in the study, providing study centre contact details, and reminding them of the time until their free month at the end of follow up.

Anyone who completes follow up (a total of 24 weeks) will be rewarded with a free month of text messaging and 100 CNY mobile phone based payment (whether they quit or not), a reminder of these benefits will be sent to the participants at the beginning of the study and each month. All participants will be checked at 4, 8, 12, 16, 20, 24 week points about continuous smoking abstinence, point prevalence of abstinence, how many cigarettes per day during the last 4 weeks and past 1 week if they are still smoking, levels of craving, weight and so on. Symptoms of anxiety and depression, sleeping quality will be only asked at before quit day, 8 week, and 24 week points, and a phone call will be made for their significant other (e.g., family member, friend) to make a further confirmation about their quit status.

### Measures/Instruments

Recruitment rate: whether we will be able to recruit our target sample size of 200 participants (for the pilot study) in the time allotted in the project, which will be within 3 months. Retention rates will be measured in two ways: (a) within the intervention, whether participants would respond to queries about their smoking status; and (b) in the research, whether we could achieve follow-up rates high enough to conduct a larger randomized control trial (at least 75 %). Performance of the software that sends the program text messages (i.e., the messages needed to be sent successfully to all mobile phone carriers as designed).

### Quit messages

Types of Quit message show in Table 1 and Database of cessation messages show in Table 2.

**Table 1:** Types of message for ‘happy quit’ program

Message type	Description	Examples
Invitation message	Describes the ‘happy quit’ program.	If you are an adult smoker intending to quit, please join our free SMS supported quit smoking activity. This program funded by CMB. This approach has been proven to be successful in some countries.
Preparing to quit	Describes steps and ways to take in preparing to quit smoking.	(1) Congratulations!! The hardest part—deciding to quit—is already behind you. Write down all of the reasons why you want to quit. (2) Before quitting, you should track your smoking; Try to cut down by 25 %, that is from 20 to 15 cigs per day. We’ll check in later. (3) Time for a Mini-Quit challenge. For the next 4 h, stay away from cigs. Practice dealing with cravings without smoking. We’ll check in 4 h.
Benefits of quitting	Describes the health-related, social and financial benefits of quitting.	(1) Figure out how much money you spend on cigarettes every year. What else can you do with that money? (2) For healthy and fresh breath, beautiful appearance, please keep on quitting! (3) There are so many benefits to being smokefree, such as looking well, having fresh breath, a good appetite and being full of energy. Tell your friends what you look forward to most! (4) Quitting smoking is the only way to stop damage to your body caused by smoking.
Seek for help	Describes seek for help to deal with crave, mood and reasons for quit.	Get more support whenever you need it by texting 7 (CRAVE), 8 (MOOD), 9 (why quit) at any time.
Coping and coping strategies	Describes and encourages the effectiveness and use of cognitive and behavioral strategies to avoid smoking during a craving or impulse to smoke.	(1) Treat every day like your quit day. Pretend like it is the first day without cigarettes and be prepared for temptation. (2) Don’t let things get you down. Your journey to smoke-free might be a struggle, but looking back will be amazing. Try to relax. Find an activity that is relaxing to you like listening to music, walking or taking a hot bath. (3) Think positively and find healthy outlets for stress or anger. If something bothers you, learn to relax quickly by taking deep breaths. Take 10 slow, deep breaths and hold the last one. Then breathe out slowly.
Discomfort and difficulties	Discusses discomfort associated with the quitting process and how the participant may see his or her discomfort as normal and how to cope with such discomfort	(1) Cravings become weaker every day that you don’t smoke. It’s harder in the beginning and will get easier with time. (2) Cravings last less than 5 min on average. Even the strongest cravings will go away after a few minutes. Focus on something else and remind yourself why you are smoke-free. (3) Drinking a lot of water will fight off cravings and help to keep you hydrated. (4) Try using sugarless gum, mints, toothpicks, straws or sunflower seeds to help keep your mouth busy.
Encouragement	Offers motivation and support to continue with quitting.	(1) 24 h smokefree! That is a MAJOR milestone! Be sure to reward yourself. Give, say or do something nice for yourself to celebrate your success. (2) 5 days smokefree! Smoking dulls taste buds. Luckily, your taste buds regenerate after several days without cigarettes. Treat yourself to a nice meal. (3) We know quitting is tough but stay strong! We all have bad days, and you will get through this. Do whatever to boost your mood-just don’t smoke.
Relapse	Describes how to prevent slips and the norms of slipping and how to get back on track; Clarifies reasons for quitting and to recommit; and teaches the participant to learn about what didn't work and new strategies	(1) You have come so far. Be careful when you go to a party, game or bar-do not let yourself slip. (2) You may not always feel as confident as you should and might slip. Keep trying no matter what. It Is possible to quit for your health. (3) How did you feel just before you smoked? Recommit to complete quitting—cutting down isn’t good enough for you! (4) Most smokers try to quit 6–7 times before they quit for good. Don’t quit quitting!

**Table 2:** Database of cessation messages (will be modified during the pilot study)

Message type	Database of messages per quitting stage					
	Pre-quit	Quit day and day 2	Early quit	Late quit	Relapse	Encouragement to try quitting again later
Preparing to quit	170	0	0	0	0	0
Benefits of quitting	40	10	30	190	30	30
Coping and coping strategies	140	20	110	60	70	20
Discomfort and difficulties	10	30	30	10	20	0
Encouragement	30	10	50	30	50	30
Quitting skills	20	0	0	60	0	0
Relapse	0	0	0	0	50	0
Total (1350)	410	70	220	350	220	80

### Outcome measures

**Self-reported continuous smoking abstinence**: no more than five cigarettes smoked in the past week at 4 weeks follow-up and no more than five cigarettes smoked since the start of the abstinence period at 6 months of follow-up. **Point prevalence of abstinence**: not even a puff of smoke, for the last 7 days, at 4, 8, 12, 16, 20 and 24 weeks. Abstinence will be assessed by means of brief telephone interviews at each time point. **Cigarettes smoked per day:** number of cigarettes smoked per day within the past 4 weeks and the approximately total number of smoked cigarettes within the past 4 weeks.

### Independent variables

Characteristics of cigarette smokers: age, gender, marital status, education years, smoking duration, severity of nicotine dependence, weight, sleep quality, levels of anxiety and depression et al. These will be collected by phone call and using a series of questionnaires, such as questionnaire of demographic characteristics, Pittsburgh sleep quality index, state-trait anxiety inventory, Beck depression rating scale, Fagerstrom Test for Nicotine Dependence.

### Statistical methods

**Power estimation:** On the basis of the results of the recent review paper [20] and the large sample size study published in the Journal of Lancet [15], we estimated that the control group quit rate would be around 5 % (ranged from 4.9 to 27.6 %) and loss to follow-up will be 10 %, with 95 % as an accepted standard for statistical significance. According to a power analysis, only 486 participants will be required in this group. However, given that none of the study from China, we plan to assess 4000 participants, and 2000 participants (1000 participants in each group) will be eligible and randomize to intervention or control groups. With 10 % loss to follow-up, we will have a 90 % of participants available for analyses, which will provide sufficient power to detect a difference.

**Comparison between Intervention group and control group:** For the data, *t* test, Chi-square test and logistic regression will be used for the comparison of the self-reported continuous smoking abstinence, point prevalence of abstinence, cigarettes smoked per day between the two groups. Repeated measures analyses of variance will be used for the comparison of levels of craving, weight, sleep quality, levels of anxiety and depression between the two groups. An alpha level of 0.05 (two-tailed) will be chosen for all statistical tests in this study. For the primary analysis we will use multiple imputation, which uses the observed predictors of outcome and the predictors of loss to follow-up to impute missing outcome data, thus attempting to correct for any potential bias caused by it (using the intent-to-treat principle, missing will be counted as smoker).

### Pilot study

**Fig. 3:**
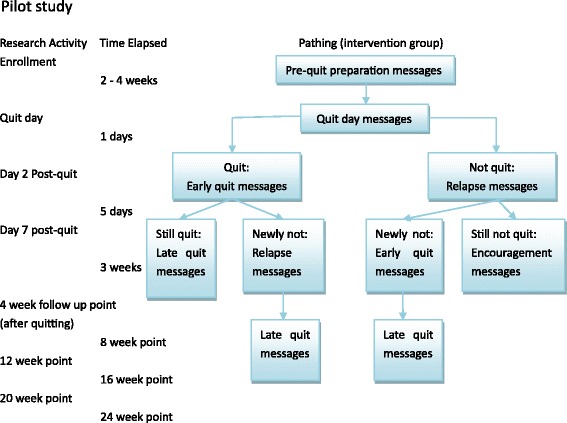
Development of ‘Happy Quit’ text messaging program flow. Control group: participants from control group will only receive one text message every week, thanking them for being in the study, providing study centre contact details, and reminding them of the time until their free month at the end of follow up

**Fig. 4:**
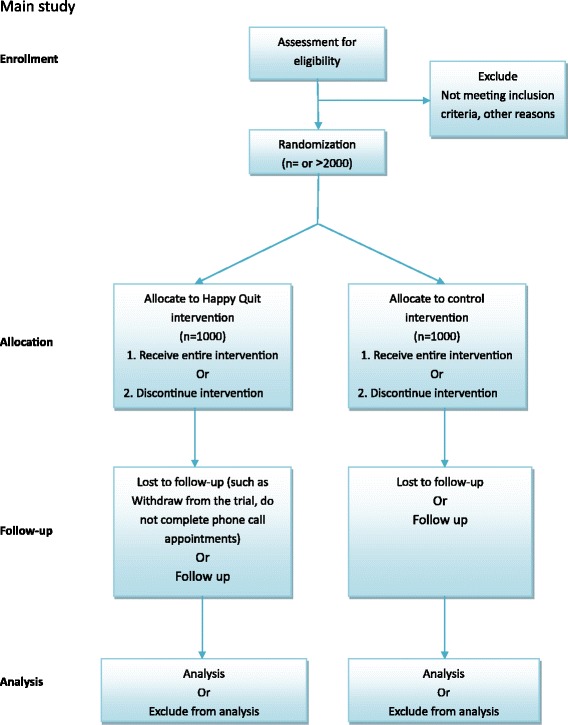
Flowchart of program profile

The following research activities with 10 % of the whole sample size (about 200 participants) will be conducted during pilot study. Flowchart for the pilot study shows in Fig. 3 and main study in Fig. 4.

**Develop the ‘Happy Quit’ program:** Development of smoking cessation text messages (detailed can be seen in Tables 1 and 2.) will be based on previous studies in other countries, and smokers and smoking cessation professionals in China. Questions for assessing program acceptability show in Appendixes (page35). It will take two to three months to develop the program.

**Assess the efficacy of ‘Happy Quit’ intervention:** The pilot study will be implemented with approximate 200 participants (100 participants in intervention group and 100 participants in control group), and feedbacks (such as acceptability of smoking cessation text messages and number of messages/day) from experts, providers and participants will be collected to improve the quality of ‘Happy Quit’ program. In this single-blind, 24-week, randomized trial, Participants will be allocated to either a control group or to a group that receives a supportive smoking cessation program.

**Examine the feasibility and acceptability of ‘Happy Quit’:** The feasibility and acceptability of the ‘Happy Quit’ program will be examined during the whole period of the pilot study stage. Examinations will include test-retest reliability and internal consistency of the instruments, acceptability of the message contexts, significant differences of intervention efficacy among smokers with different age and educational level, and decreased number of smoked cigarettes after intervention.

### Study status

Library of short messages for ‘Happy Quit’ program has be built, which began to develop in January 2016. Recruitment and data collection for this study will begin in April 2016 and will continue for the next two years.

## Discussion

Tobacco use is a major cause of disease burden and the number one preventable cause of death in the world. About one-third of the world’s tobacco is produced and consumed in China. Among the general population of smokers, most report a desire to quit. Unfortunately, the availability of smoking cessation programs in China is extremely limited, and the majority of cessation attempts end in relapse. The rapid development of mobile health technology (mHealth) provides unprecedented opportunities for improving health services and reaching underserved populations, and the effectiveness of text message mobile phone program on improving smoking cessation has been reported in other countries.

With relative large sample size, an mHealth Smoking Cessation Trial for individuals who subscribed to Nokia Life Tools showed that text message–based smoking cessation intervention can be successfully delivered in China and is acceptable to Chinese smokers [21]. However, this study had high opt-out rates and low postintervention response rates. Another study, relatively small sized sample (92 participants in the intervention group and 87 participants in the control group), evaluated the effectiveness of a mobile phone text-messaging based smoking cessation intervention package among Chinese adolescent smokers aged 16–19 years from six schools, which showed that the interactive and tailored assistance provided by the mobile phone text-messaging was effective in smoking intervention in Chinese adolescent smokers [22]. In this proposal, interactive and tailored will be provided for adult smokers. To the best of our knowledge, this is the first large sample size trial using interactive and individualized mobile phone-based text message interventions for adult smoking cessation in China. This proposed study could have widespread implications for tobacco control and health promotion in China, and can provide valuable experiences involving smoking cessation in China. We will submit a report to the Chinese Center for Disease Control and Prevention (China CDC), and distribute detailed reports on program development, intervention instruments and results of our evaluation to other administrations and organizations who are interested in promoting tobacco control in China.

However, there are some limitations in this proposal: First of all, this proposal will not provide biochemical verification for self-reported claims of abstinence due to the geographic spread of the sample, cost and other practical concerns. However, false reporting is considered to be minimal when there is little or no personal contact between treatment provider and participants (i.e. only through telephone); hence, it not likely that misreporting could compromise conclusions. Furthermore, the biochemical tests are not perfect. Cotinine has an in-vivo half-life of about 20 h, and can only be detected with a cotinine test for a few days after the use of tobacco. Carbon monoxide can be detected only for about 24 h after tobacco use. Secondly, although efforts will be made to ensure that the research staff remain be masked to whether a participant will be in the intervention or control group, occasionally trial participants would reveal this information to the study staff. This information could bias our estimates of self-reported abstinence. Thirdly, participants who will be allocated to the control group could reduce their motivation to quit. In order to address this issue, the control group will be recommended to receive smoking cessation care once the study is over. Despite those limitations, the expected benefits of this research project are numerous, such as providing interactive and individualized mobile phone-based text message smoking cessation program, developing suitable mhealth model in China, promoting mhealth in smoking cessation and other mental disorders’ treatment and prevention.

## Abbreviations

mHealth: The use of mobile information and communication technologies for improving health
SMS: Mobile phone based short text messaging service
